# Barriers, facilitators, and disparities in retention for adolescents in treatment for substance use disorders: a qualitative study with treatment providers

**DOI:** 10.1186/s13011-020-00284-4

**Published:** 2020-06-18

**Authors:** Andrea Acevedo, Nellie Harvey, Maureen Kamanu, Shalini Tendulkar, Sasha Fleary

**Affiliations:** 1 Department of Community Health, Tufts University, 574 Boston Avenue, Suite 208, Medford, MA 02155 USA; 2 Eliot-Pearson Department of Child Study and Human Development, Tufts University, Medford, USA

**Keywords:** Substance use treatment, Adolescents, Retention, Disparities

## Abstract

**Background:**

Retention in substance use treatment is one of the strongest predictors of improved outcomes among adolescents, making retention an important goal of treatment. We examined treatment providers’ perspectives on barriers and facilitators to treatment retention among adolescents, and their views on contributors to racial/ethnic disparities in retention including ways to address disparities.

**Methods:**

Semi-structured interviews were conducted with 19 providers at state-licensed detoxification, residential, and outpatient facilities serving adolescents for substance use disorders in Massachusetts. Interviews were coded by at least two independent coders.

**Results:**

Providers identified barriers and facilitators at the policy/systems, facility, family, and client levels. Some of the barriers included insurance limits on sessions/length of stay and low reimbursement (policy/systems), staff turnover (facility), low family engagement (family), and low internal motivation (client). Some facilitators mentioned were support from state’s substance use agency (policy/systems), flexibility with meeting location (facility), family participation (family), and high internal motivation and presence of external motivators (client). Barriers that contributed to racial/ethnic disparities included lower socio-economic status, language barriers, and mistrust. Having bilingual/bicultural staff and multi-lingual materials, and facilitating transportation were identified as strategies for reducing disparities in treatment retention.

**Conclusions:**

It is critical that adolescents who access substance use services remain and complete treatment and that there is equity in treatment retention. Provider perspectives in factors associated with retention can inform the development of comprehensive interventions and policies to help improve retention and reduce disparities.

## Background

Adolescent substance use and substance use disorders (SUD) are important public health concerns given their impact on youths’ physical and mental health and their associated societal costs. Adolescents who use substances experience a more rapid progression to addiction than adults and most adults with a SUD report initiating drug use during adolescence [1]. In 2017, almost one million adolescents aged 12 to 17 had a SUD, representing 4% of the total adolescent population in the United States [2]. Adolescents with a SUD are more likely to make risky decisions such as engaging in unsafe sex and driving under the influence. They also experience problems in familial and peer relationships, academic issues, a loss of interest in healthy activities, impaired memory, mental health problems and an increased risk of overdose [1]. These short- and long-term repercussions suggest the importance of addressing substance use disorders at an early age.

Substance use treatment can help reduce substance use and improve social and school functioning [3–5]. However, only about 9% of adolescents with a SUD received specialty treatment in 2017 [2]. The gap between those who need treatment and those who access treatment was even higher among some adolescents of color. Compared to White adolescents, Black and Latino adolescents with SUDs had lower rates of treatment access [6, 7].

Among clients who receive treatment, treatment retention is one of the strongest predictors of improved outcomes, making treatment retention an important goal of treatment [4, 8, 9]. Yet, at the national level, about one-third of adolescents leave treatment before completion [10]. Multiple factors influence retention among adolescents once they enter treatment. At the client level, adolescents who have low treatment readiness, those whose primary substance is alcohol or marijuana (as opposed to “harder” drugs or polydrug use), clients who experience emotional problems from substance use, and those with a trauma history are at increased risk of dropping out of treatment [11–13]. Family and parental characteristics also impact treatment engagement and retention; adolescents are more likely to stay in treatment if their parents recognize their substance use as a serious problem [14] and have higher expectations for their child’s potential educational attainment [11, 14]. On the other hand, adolescents not likely to stay in treatment are more likely to report living with only one parent and a history of familial substance use [13]. Having commercial health insurance (compared to no insurance or Medicaid) has also been associated with lower treatment retention [13].

Few studies have examined racial/ethnic disparities in treatment retention among adolescents. Studies examining racial/ethnic differences generally found that Latino and/or Black youth had lower treatment retention compared to their White counterparts [15, 16]. Nationally, Black and Latino adolescents receiving treatment in publicly funded facilities had significantly lower treatment completion rates than non-Latino White adolescents, and these differences were not explained by greater severity of symptoms [17]. For disparities in treatment access among youth, Alegria et al. have conceptualized federal and economic health care policies, operations of the facility, environmental context, and client level factors as potential mechanisms [7]. There is some evidence of these factors playing a role in treatment retention as well. The social context, such as racial/ethnic composition of the area and regional differences in financing of adolescent treatment, explained a substantial proportion of the Black-White and Latino-White treatment completion gap among adolescents [17]. Socio-economic status (SES), measured as unemployment, educational attainment, and homelessness largely explain racial/ethnic disparities in treatment completion among adults [18], but these measures are not as relevant for youth as most are still in school, and less than 1% are homeless when they enter treatment [10].

Most of the research on adolescent treatment retention and the limited research on disparities in retention among adolescents does not incorporate the voices of substance use treatment providers. Provider perspectives are needed to fully understand the factors influencing treatment retention and to inform the development of comprehensive interventions to help improve retention and reduce disparities. The purpose of this study was to explore treatment providers’ views on barriers and facilitators to treatment retention among adolescents, as well as their views on factors influencing racial/ethnic disparities in treatment retention among this population. We used the TCU Treatment Process model to guide our analysis in understanding factors associated with treatment retention among adolescents [19]. This model was created to understand the processes, including early engagement and treatment retention, in substance use treatment and interventions at different stages of treatment that lead to better outcomes. In this model, client (e.g., treatment readiness, addiction severity) and facility (e.g., staff, climate) attributes at treatment entry influence the level of participation early in treatment, and social support contributes to longer retention after early engagement. We extended this model to include an additional focus on understanding racial/ethnic disparities in treatment retention.

## Method

### Setting

We interviewed providers of facilities serving adolescents for SUDs in Massachusetts that were licensed by the state’s Department of Public Health (DPH). Providers in detoxification/stabilization facilities, residential treatment facilities, and outpatient treatment facilities were included in this sample. Detoxification/stabilization (referred to from here on as “stabilization”) facilities provide a short-term treatment environment to assist with medical monitoring, emotional stabilization, and eventually, referrals for aftercare. Residential programs are geared towards youth experiencing health, emotional, familial, or social problems from their substance use that require intensive live-in care. Outpatient programs provide less intensive care and deliver counseling and psychoeducational services for SUDs, with usually one session every week or every other week.

### Study design and recruitment of Participant*s*

We used semi-structured qualitative interviews to understand providers’ views on barriers and facilitators to treatment retention, as well as their views on reasons for racial/ethnic disparities in treatment retention among adolescents. The interviews also contained questions related to barriers, facilitators, and disparities in access to treatment, but here we focus on the data related to retention only.

We developed a list of adolescent treatment programs through the Massachusetts Department of Public Health website and the SAMHSA treatment locator. We used purposive sampling to reach out to specialty treatment providers that served adolescents from a variety of regions within Massachusetts. Special efforts were made to recruit providers from stabilization and residential facilities as only a few such programs exist in Massachusetts. Whenever possible, program directors were identified via program websites or contacts prior to recruitment. Directors were contacted by the first author via phone or email using a standardized script which described the study and asked for their participation. In some cases, the first author was referred to a clinical director or clinician more knowledgeable about retention issues in the facility.

Seven interviews were conducted in 2015. Because after preliminary analyses tracking barriers and facilitators it was clear that we had not reached saturation (new information was being gathered through the last interview). Therefore, we conducted additional interviews in 2018. Each participant contacted in 2015 was offered a $35 gift card as compensation for their time. We increased the amount to $50 for interviews conducted in 2018 as that seemed more appropriate for the time required to participate and with the availability of new funding. A total of 25 program directors were contacted and 19 agreed to participate (76% response rate).

### Data collection

Prior to the interview, participants completed a brief survey about their demographic characteristics and work experience, as well as information about their facility including ownership, focus, level of care provided, average number of clients served annually, and client demographics. Semi-structured interviews examined providers’ perspectives on: 1) facilitators to adolescents remaining in treatment longer, including what their facility has done to successfully support retention among youth, 2) barriers to treatment retention or factors associated with adolescents leaving treatment early, and 3) potential factors associated with lower retention among adolescents of color, and how needs of adolescents of color are or could be addressed to reduce disparities in retention.

Seven interviews were conducted in 2015 and twelve in 2018. All interviews were conducted in person at the treatment facility in a private space. All interviews were conducted by the first author, a researcher in substance use treatment with training and experience in conducting research interviews. Interviews took between 20 min to an hour, with additional time allotted for informed consent procedures and completion of the survey.

### Data management and analyses

Most interviews were audio-recorded. One participant did not consent to be audio-recorded, and one interview was only partially recorded due to a recorder malfunction. In these cases, detailed notes were used. Interviews were transcribed verbatim; transcripts were uploaded into NVivo software, Version 12 [20]. The interview data were analyzed using thematic analysis [21]. Because issues related to treatment retention were assumed to vary by type of services provided at the facility, the coding was done in stages in this order: stabilization, residential, and outpatient facilities.

A preliminary codebook was developed by three research team members using a hybrid deductive and inductive approach to code development [22]. First, deductive codes were identified based on the study questions and aims: facilitators to retention (including what the facility has done to successfully improve retention), barriers to retention, reasons for disparities in retention, and ways to address disparities in retention (including what the facility has done to address disparities). Then, the three research team members independently reviewed one of the transcripts, conducted a line-by-line reading of the data, identified inductive codes within the data, met to discuss the coding and compiled the codes into a more expansive codebook. This codebook was then used to recode the original transcript. The three research team members then independently coded three additional transcripts. After each transcript was coded, the three research team members met to compare their coding. Whenever divergent interpretations occurred, the transcript was re-reviewed, and coders discussed discrepancies in coding until consensus was reached. Throughout the process of coding the first four transcripts, new codes that emerged were entered in the codebook. The codebook was applied to the remaining interviews; each was coded independently by two of the coders who subsequently met to discuss and resolve any discrepancies, and if necessary, add new codes. The transcripts that had already been coded were reviewed using the final codebook.

We then used the TCU Treatment Process model to come up with initial themes from the subcode categories [19]. Based on the data, final categories for barriers and facilitators were grouped in broader themes and were reviewed by the entire research team for logic.

## Results

### Participant and facility characteristics

Nineteen providers participated in the study. Table 1 shows participant characteristics and characteristics of their facilities. Most participants were directors (executive or clinical directors). Participants had an average of 11.2 years of experience in the substance use treatment field and had worked an average of 5.5 years in their current facility. Most participants were White (12 women and 4 men), and four participants were people of color. Participants worked in 19 different facilities, most of which were outpatient. All facilities were private not-for-profit, and most were community-based, although three of them were outpatient facilities based in hospitals. Most facilities were adolescent-specific, and four of them (all outpatient) provided services for adolescents and adults. The number of clients served annually ranged from approximately 30 to about 650, with larger number of clients served by the stabilization facilities.

**Table 1:** Participant and Facility Characteristics

Characteristic	N	Mean (range)
***Providers-Total***	19	
**Titles**		
Executive/Program Director	11	
Clinical/Medical Director	5	
Clinician	3	
**Race/ethnicity and gender**		
White women	12	
White men	3	
Black women	1	
Black men	1	
Latinas	2	
**Years in SUD treatment field**^1^		11.2 (1–35)
**Years working in the facility**^1^		5.5 (0.5–20)
***Treatment Facilities--Total***	19	
**Level of care**		
Detox & Stabilization	2	
Residential	4	
Outpatient	13	
**Hospital based (Outpatient)**	3	
**Focus**		
Substance use only	5	
Mental health only	1	
Substance use and mental health	12	
Integrated primary care and behavioral health	1	

**Notes:**^1^N = 18 due to missing data

### Themes

We used the TCU treatment process model to guide our analysis [19]. We adapted the model based on the themes that emerged from the data in two ways. In the model, client’s family factors are included as “social support”. We used “family” instead because family emerged as a strong theme on its own, and it was not described as social support. Furthermore, providers mentioned several barriers and facilitators at the systems and policies level, and these factors are not included in the model. The final themes for barriers and facilitators were: 1) client level barriers/facilitators, 2) family level barriers/facilitators, 3) facility related barriers/facilitators, 4) policy/systems barriers/facilitators. Providers offered less varied information on disparities. Therefore, we include those findings on their own, without categorizing them under the four themes.

### Barriers to treatment retention

Table 2 includes the list of different barriers within the major themes. Below we describe themes that were most salient, identified by several providers. To protect confidentiality, we did not differentiate between stabilization/residential facilities as there were only two stabilization facilities in the state at the time of data collection.

**Table 2:** Barriers to adolescent treatment retention

**THEMES AND SUBTHEMES**	
**Family-related**	
Limited family participation/visits	
Family members use substances	
Other^1^: costs associated with treatment, adolescent is caretaker, violence in the home	
**Policy/Systems**	
Health insurance barriers	
Inappropriate placement	
Other^1^: Transportation system	
**Client**	
Lack of motivation/readiness	
Difficulty adjusting to rules/structure	
Trauma history/mental health comorbidity	
Younger age	
Other^1^: Withdrawals symptoms at treatment entry, marijuana as main substance, run away	
**Facility**	
Staff burnout /turnover	
Other^1^: Non-smoking policies, following traditional model of talk therapy/coming to clinic, program location	

*Note:*^1^Mentioned less frequently

#### Family level barriers

Many providers mentioned family factors associated with lower treatment retention among adolescents, particularly limited family engagement. For those in stabilization/residential treatment, lack of family visits was specifically mentioned as a barrier to adolescents staying in treatment:

*One thing that’s a big struggle is if a kid does come here from the [city], parents not coming out to see them. Which, that right there, is a little bit harder to keep them –– you know, engaged in services, or keep them enrolled or admitted, because no one’s coming to see them.****P18-Stabilization/Residential***

It is important to note that providers often said that families experienced their own barriers to being involved in treatment. These barriers included transportation problems, lack of child-care, and work conflicts.

*Sometimes, the parents can’t make it. Sometimes, it’s just difficult for them. Sometimes it doesn’t work with their schedule or transportation, so other things come in play… Here, it’s sometimes lack of resources for the parents if they have more than one kid and they don’t have anybody to look after the kid to be able to bring the other ones to therapy. That’s been identified as something, in the past, that’s been a barrier.****P10-Outpatient***

In some cases, the lack of family engagement was seen as a sign of families not being invested in the youth’s treatment, which, according to participants, contributed to lower treatment retention. As one participated stated:

*So, the kids who come in, and their families are like, “this is their problem we’re just sending them”. They’re going to be struggling with completing and completing it successfully.****P12-Outpatient***

Another family level barrier was related to family members using substances themselves.

*So, when we have parents who are actively smoking marijuana but sending their kid here and saying, “you need to get treatment for this” and they’re still drinking and smoking. We’re like “it’s really tough for us to support you in setting that expectation when you’re not kind of following it right now for yourself”.****P12-Outpatient***

#### Policies/systems

Many providers mentioned systems or policy level barriers such as issues with health insurance negatively impacting treatment retention. Some providers said they often had to ask insurers for multiple authorizations to cover needed treatment sessions or longer stays; efforts which were time consuming and not always successful, and which contributed to lower retention. Providers also said that insurance plans do not reimburse enough or cover many of the activities that providers do when working with adolescents. For example, providers expressed frustration that insurance companies do not pay for the time they spend accompanying the youth to court, assisting with job searches, attending meetings at their clients’ schools, traveling to meet clients off-site, or for the costs associated with prosocial activities. These practices were seen as critical to help youth remain in treatment, and are part of evidence-based protocols for adolescent treatment, such as the Adolescent Community Reinforcement Approach (A-CRA) [23].

*You bill everything, but you’re never able to fund the program on your billing… Well, it [reimbursement] doesn’t cover it; doesn’t cover what it is. And it tends to fragment what you do. I think we billed for case management to some insurer. I don’t even think we got paid… And then it’s like you’re going to get $10 and it takes you $25 to do the billing.****P9-Outpatient***

*… a lot of times insurance won’t pay for you sitting with them in a court hearing, even though it’s important, but that’s not something that they would necessarily cover … Like an A-CRA procedure, one of them is job seeking, and a lot of them won’t let you pay for that if you’re going out in your car picking up applications.****P1-Outpatient***

These challenges experienced with health plans contributed to staff turnover, which also was mentioned as a retention barrier (see section 3.2.4). Other insurance-related barriers mentioned by providers were high deductibles and discontinuation of Medicaid coverage.

An additional “systems-related” barrier, mentioned by providers in residential and stabilization facilities, was inappropriate placement. Inappropriate placement was described both as 1) an adolescent having serious behavioral or mental health issues that the treatment facility did not have the capacity to address, or 2) the opposite, where a youth was referred to stabilization or residential treatment, yet, the youth’s substance use was not severe enough to warrant that level of care. Several of the examples given were referrals from the state’s child protection agency.

*If this child is in their (child protection agency) custody, they oftentimes will use our program as a placement… [they] can say, “We don’t have a place to put them”****P17-Stabilization/Residential***

*So, it’s a little bit of a system-wide kind of problem... There are not enough beds for [child protection agency] for their types of kids. … So, it’s like, “We don’t know what to do with this kid. He smokes marijuana, let’s put him in a [rehab]”****P18-Stabilization/Residential***

#### Client Level Barriers

Providers mentioned several client characteristics as barriers. Although providers had strategies in place to support treatment retention, several providers mentioned that there were some clients who were not motivated or ready for treatment, and thus, were more likely to end services early. As one provider stated:

*I always say I think that [motivation] a big predictor of anything, you know. Of anything. I talk a lot with the clinicians about we can’t work harder than the clients. And, when you see that they have lack of motivation, maybe they’re not ready.****P16-Outpatient***

Among clients in residential treatment specifically, another client-level barrier to treatment retention was adjusting to being away from home; including adjusting to new rules that come with living in a residential setting and living in a setting with other individuals with a SUD.

*Others think that going to [Massachusetts’s Juvenile Justice Agency] or lockup would be easier. And I think that's because we hold them accountable for behaviors here and expect them to make positive changes for themselves*. ***P3-Stabilization/Residential***

Many providers said that youth with trauma histories and co-occurring mental health disorders had more difficulty staying in treatment. Providers also noted lower treatment retention among younger adolescents as they were more likely to have behavioral issues, were more impulsive, and were more likely to ignore consequences.

#### Facility level barriers

Several providers mentioned staff related facility-level barriers, such as staff burnout and staff turnover. Some said that low pay and the stressful nature of the job contributed to these staffing problems:

*And a lot of times insurance requires a lot of paperwork that you don’t necessarily get paid for, and so it’s just really stressful, I think for the staff to do home based work and deal with traveling, working with a difficult population, and then also having to do all the paperwork that you have to do and not getting paid that much too, so it just makes it hard, and then you get a lot of staff turnover and that’s hard for everyone. … But I think if we paid them a little bit more based on the rates, we would be able to hold on to them.****P1-Outpatient***

Other facility related factors mentioned as barriers to youth continuing in treatment included the facility location (difficult to access), non-smoking policies, and following the traditional model of clients having to go to the clinic.

#### Barriers contributing to racial/ethnic disparities in treatment retention

Providers mentioned several barriers that contribute to racial/ethnic disparities in treatment retention. Several providers mentioned lower family socio-economic status (SES) as an underlying reason for racial/ethnic disparities in youth treatment retention. SES impacted racial/ethnic minority clients’ and families’ access to transportation. A residential provider mentioned that youth of color in residential treatment sometimes worried about being able to contribute financially to their family while in treatment. Additionally, families with lower SES may not be able to prioritize treatment due to more urgent, competing demands.

*Some families are moving from home to home and the priority is not bringing in their kid to treatment. It’s having a roof over their heads, food, clothes, and hygiene.****P11-Outpatient***

Another contributor to disparities was the language barrier experienced by many immigrant families, which limited the parents/guardians’ involvement in their child’s treatment. Some providers also thought that families of color may not know how to access community resources, and that they were less trusting of systems that support treatment retention such as the legal and educational systems. One provider said that mistrust was, understandably, particularly common in undocumented families. Finally, a provider mentioned mistrust of the treatment system itself as a barrier to retention among youth from some racial/ethnic groups.

*Some sections of the population feel like they’re targeted by law enforcement or treated more harshly through the courts… And we see it a bit here because we’re part of the system and they don’t feel empowered by it. It just tends to have a reinforcing sense that I’m not empowered in this system... I can see that happening here where they’ll be definitely less likely to continue*. ***P9-Outpatient***

Some providers expressed general concerns that they were not meeting the needs of clients of color, and some cited broader societal and policy barriers that contributed to lower retention among youth of color. For example, one provider mentioned structural inequality, which made it difficult for youth of color to access resources in general. Another provider mentioned that lack of flexibility by insurance companies in meeting location and length of treatment, affected youth of color’s retention. As one provider stated,

*A Black or Hispanic youth who doesn’t want to necessarily meet in the home, or they can’t meet in their neighborhood… you have to be more open to seeing them for longer or in a different type of environment … the reason why they [treatment facility] can’t be flexible is because insurance isn’t flexible and you need to get paid.****P5-Outpatient***

### Retention facilitators

Table 3 lists facilitators to treatment retention within the major themes.

**Table 3:** Facilitators to adolescent treatment retention

**THEMES AND SUBTHEMES**	
**Family**	
Family involvement	
**Facility**	
Engage families	
Extended hours/walk-ins	
Flexibility in meeting location	
Flexibility with tardiness/missed appointments	
Devoted, motivational, respectful staff/staff-client relationship	
Meeting youth “where they’re at” with their substance use goals	
Food/program incentives	
Sober/pro-social activities	
Communication with youth between sessions	
Other^1^:Communicate with collaterals/across agencies, recovery coach-case management, external funding/donations, use of evidence-based practices/telehealth, small program	
**Client**	
Motivation	
External motivators	
Other^1^: Extra-curricular involvement, more severe use/overdose, prior experience with treatment/sober, older age	
**Policy/Systems**	
State’s Medicaid program	
Financial support from state’s substance use agency	

#### Family level facilitators

Family involvement was commonly cited as a facilitator to treatment retention. Family involvement included taking the youth to treatment, participating in family treatment sessions, and following the providers’ recommendations. Families also positively influenced retention if they provided encouragement, if they understood that reducing substance use is difficult, and viewed treatment as “*not just an option, but a necessity*”.

Several providers said family engagement was facilitated by the family having more resources, including time and childcare.

*The families who have more means [tend] to be involved in the treatment. They don't have to work two jobs. They have a car. They have free time to take the kid to the clinic if they need to be in the clinic. Or they have the resources and in some way they're higher functioning, so they can actually be a part of the treatment process.****P5-Outpatient***

#### Facility level facilitators

Working with families and engaging them in their children’s treatment was a common facility-level facilitator of longer treatment retention.

*Ability to bring in families and support them and provide them [with] education, that’s a huge piece to it because they don’t always, a lot of times don’t understand what’s going on. They’re just ‘why do they keep doing this’… The family is engaged in treatment which means they’re getting part of treatment.****P12-Outpatient***

Having extended hours for youth and their families, such as evenings or weekends, and seeing youth on a walk-in basis was also identified as a facilitator to retention.

*They don’t fit into a 9 to 5 – because they don’t even get out of school until 3:00, 3:30. I do have people who work with kids on weekends. …the flexibility of when and where you meet adolescents is key. It’s not like with adults.****P2-Outpatient***

*We have our drop-in hours… We instituted them in large part, to help with retention.****P14-Outpatient***

Providers said meeting youth in their home, school, or in a community setting, as opposed to requiring youth to come to the facility for an appointment, helped youth remain in treatment.

*We'll go to the home. We'll go to the school. We'll see them anywhere in the community, meeting them where they are and where they're willing to meet for treatment… That ability to meet them where they are instead of asking them to come to us.****P5-Outpatient***

Several providers also said that flexibility with tardiness and missed appointments helped with retention.

*You also have to be flexible – they may show up ten minutes late or they may show up 15 minutes early. And they’re not waiting. You have to be flexible enough to say, “Come in now.”****P2-Outpatient***

*We have these patients apologize all the time for missed appointments. None of that impacts whether or not they can come back. I think that as patients believe that we’re not just saying that, it really does help with retention.****P19-Outpatient***

Providers mentioned the importance of accepting youth “where they’re at” in terms of their substance use and goal setting.

*So, we don’t have the philosophy of “you’re using you’re out, come back when you’re ready”. That’s really not a helpful model for adolescents and young adults. And, I would say, no one with substance use disorders.****P14-Outpatient***

*It's an abstinence-based group, so they have to agree to – they have to commit to abstinence… And if they do use during the course of group, it doesn't mean they get kicked out of group.****P7-Outpatient***

Providers also often mentioned staff characteristics and positive staff-client relationships as helping with treatment retention. They mentioned staff being “energetic” and devoted, and youth feeling heard and respected by the staff, as facilitators to retention.

*When they come in and they develop a relationship and they feel safe and they feel like they’re wanted and they have people to talk to. That’s the key and I think that’s really the number one thing… So if we don’t do a good job in that, we’re going to lose the kid.****P6-Stabilization/Residential***

Several providers said food and incentives were helpful in retention, as well as organizing pro-social/sober activities. Communication with clients between sessions, whether to remind youth of appointments or to check in with them, was also a strategy used by providers in outpatient settings for retention. Often, this was done with text messages.

*One of the things that I’ve been doing and it’s been working well is texting them. …I’ll text them and say don’t forget you’re going to be seeing me today after school… I can keep connected with them, because it takes no time. You text and say, “I hope the test went good today,” or whatever it might be.****P2-Outpatient***

#### Client-level facilitators

At the client-level, providers mentioned client’s self-motivation as a treatment facilitator; both motivation to reduce or stop using substances and motivation to be in treatment. Often, providers also described this as “readiness” or being in the right “stage of change”. External motivators were also mentioned as facilitators to treatment retention and included involvement of the state’s Juvenile Justice agency (the Department of Youth Services or DYS) (e.g., if treatment was a requirement of probation), as well as parents, school, or involvement with the state’s child protection agency (the Department of Children and Families or DCF).

*I think a part of what gets them to stay here, is that backing of, "Hey, probation wants you to complete this program.’ …A lot of them, unfortunately, come in with those charges, so that does kind of help them. Or if DCF is saying, ‘Hey, if you don’t complete this treatment, you’re going to end up in DCF care.’ So, that external factor does help with certain populations.****P18-Stabilization/Residential***

#### Policy/systems level facilitators

At the systems and policy level, providers mentioned the state’s substance use agency Bureau of Substance Addiction Services (BSAS) and Massachusetts’ Medicaid program (MassHealth), as facilitators to treatment retention. Several providers mentioned the flexibility of MassHealth in terms of “generous” coverage and a better “understanding of the needs of clients”. Some providers mentioned that MassHealth’s program “Prescription for Transportation”, a program that pays for non-emergency transportation to and from appointments for medical necessities, helped clients and their families keep appointments.

Several providers also mentioned financial support received from the state substance use agency helped with retention, as the agency often covered services that private health plans would not:

*So BSAS allows us to also bill for what they call collateral contact, so they can call the doctor and talk to the psychiatrist about the medication and things like that. They’ll pay for the clinicians’ time for that… it gives the clinicians credit for all that other stuff that therapists do all the time that we don’t get paid for.****P16-Outpatient***

#### Addressing racial/ethnic disparities in treatment retention

When asked about what promotes retention of youth of color, providers focused on staffing: having bilingual staff to be able to engage family members who were not fluent in English and having bicultural and racially diverse staff that are “representative of the population” they serve. Several providers also mentioned working towards implementing the National Standards for Culturally and Linguistically Appropriate Services (CLAS) in Health and Health Care [24] and providing cultural competency training. Although, one provider said “*You can attend a lot of trainings, but that doesn’t mean that you’re a professional in their culture” (****P1- Outpatient).***

Several providers said it was important to have access to interpreters (if they did not have bilingual staff) and to translate their materials. Some providers expressed concern about relying on the youth for interpreting between clinician and family members, or on bilingual clinicians to translate materials.

*It’s also hard because you don’t want that clinician to be translating papers either. That’s not what they were hired for, so you’ve got to respect that they have a skill and you don’t want to abuse it.****P1-Outpatient***

Several providers said that they try to make their facilities’ environment welcoming to different groups, whether by including signage in different languages, having pictures that include different racial/ethnic groups, or having cultural celebrations. Some providers felt that being known and trusted in the community helped with reducing disparities in retention.

*I think all of our community-based work definitely addresses any disparities because we can better understand what the needs are…Coming to people who can't come to us for whatever reason. It's easier to break down the barrier of trust when you're actually in their community.****P5-Outpatient***

Finally, some providers said that providing or facilitating transportation would also be helpful in addressing disparities in retention.

## Discussion

Substance use treatment providers serving adolescents identified retention barriers and facilitators at the family, client, facility, and systems/policy levels, as well as factors associated with racial/ethnic disparities in retention. Providers are important stakeholders in adolescent substance use treatment outcomes, and this is one of the few studies incorporating their perspectives on treatment retention among this age group. Few studies have examined factors associated with racial/ethnic disparities in treatment retention among youth, and to the best of our knowledge this is the first study to examine the perspectives of providers on disparities in treatment retention with any age group.

Several client-level barriers and facilitators such as client’s internal motivation/readiness for change, external motivators, age, and mental health comorbidities are consistent with previous research [11, 12, 14, 25–28]. Providers may want to consider the treatment engagement level of such clients and address the specific needs of younger adolescents, those without external motivators or high levels of internal motivation, and those with comorbidities.

Providers identified family involvement and participation in treatment as key for treatment retention among youth, and lower family participation as a barrier, also consistent with prior research [28, 29]. Providers working with adolescents can consider several evidence-based treatment approaches which incorporate the family (e.g., Adolescent Community Reinforcement Approach; A-CRA), or are family-based (e.g., Multidimensional Family Therapy) [30].

It is important to recognize that providers noted that lower family engagement was sometimes due to structural barriers related to lower SES, such as problems with transportation, lack of childcare, and competing demands. Providers should make efforts to address those barriers and provide opportunities for all families to engage in treatment. For example, to successfully engage families from lower income backgrounds, providers might hold meetings with family members over the phone or via video conference, provide childcare, and/or have hours in the evening or weekends—some strategies that providers in this sample said they had implemented to engage families. Some of these wrap-around services require additional funding, and state substance use agencies and insurance companies could provide funding or reimbursement for these activities and services as family engagement is critical in adolescent treatment and is considered an evidence-based practice.

Providers also mentioned families seeing addiction as the adolescents’ problem and blaming youth for their substance use disorder as a barrier to retention. They also noted families understanding that recovery is difficult and providing youth with encouragement helped with retention in treatment. Treatment programs might want to include family education about the causes of substance use disorders as part of treatment. This, in turn, might help increase family support in treatment.

Providers mentioned several facility-level facilitators to retention. Our findings suggest that whenever possible, treatment programs should incorporate flexibility with lateness and missed appointments, accept walk-ins, and have evening hours [29]. Additionally, many of the facilitators to retention that were identified by providers were related to flexibility in other areas, particularly meeting youth in the community and meeting youth where they are regarding their goals surrounding substance use. Some of these strategies are part of the A-CRA protocol, and several are considered promising practices by the National Improvement on Addiction Treatment (NIATx) [31]. NIATx’s website includes several publicly available resources for testing some of these strategies using process these strategies using process improvement and could be used by providers to help improve retention among youth.

Providers also identified several barriers and facilitators to treatment retention for this age group that have not been commonly identified in the treatment retention literature, particularly those at the systems/policies level. For example, providers mentioned that health plan limits on treatment sessions/lengths of stay hindered retention among adolescents. They also said that the large amount of administrative work, the limits on what work is billable, and low reimbursement rates indirectly influence retention through staff burnout. Although insurance barriers are commonly cited as impacting access to treatment [32], our findings suggest that limits on types of services covered and reimbursement rates impact length of time that adolescents remain in stabilization services or in treatment once they have already accessed those services. Insurance companies should work to reduce the amount of administrative work required and restrictions on location of services, and eliminate the limits placed on treatment which might be inconsistent with national policies under the Accountable Care Act and parity laws.

Furthermore, providers also expressed frustration with lack of funding and reimbursement for travel time and non-clinical activities (e.g., accompanying youth to court) that providers see as important aspects of treatment and necessary for treatment retention. Insurers and state agencies that provide funding for youth treatment should make these activities billable. Insurers’ policies about location of treatment was also named as a barrier, particularly for adolescents of color. Not surprisingly, several of the facilitators they identified, such as the state’s substance use agencies and Medicaid program, as well as external funding, addressed some of these barriers. State policy makers from other states should consider allocating funding for these services through Medicaid and/or their state substance use agencies that contract to providers to improve quality of treatment and treatment retention.

Finally, a surprising systems-related barrier was inappropriate placement of some adolescents in residential and stabilization facilities, either because the youth had severe behavioral or mental health issues and/or substance use was not severe enough to require this level of care. These issues warrant careful screening at intake to determine appropriate placement. They also might point to a systems-wide problem of not enough beds in youth mental health facilities or enough foster care placements, placing pressure on substance use treatment programs to admit them. Leaders from state substance use, mental health, and child protective state agencies; treatment programs; and policy makers should work together to address this issue.

Providers focused on lower SES and language barriers as the main issues contributing to lower retention among youth of color. SES is also a major contributing factor to racial/ethnic disparities in adults [18]. Other factors associated with disparities in treatment among adults, such as substance type, treatment setting, and severity of use were not mentioned by treatment providers [18, 33, 34]. SES and race/ethnicity are deeply intertwined in the U.S., and particularly in Massachusetts where 29% of Black children, 36% of Hispanic children, and 11% of Asian children live in poverty compared to 7% of White children [35]. Thus, it is not surprising that providers would associate lower SES with racial/ethnic disparities in treatment retention. Our findings suggest that one way in which SES impacts treatment retention in youth is through family members’ inability to engage in their child’s treatment. Programs might be able to address some of these barriers by providing wrap-around services for families.

Even with additional family support services, however, families with limited resources might not be able to prioritize treatment due to other structural inequalities and urgent competing demands, such as food insecurity and unstable housing. Some providers discussed structural inequality, mistrust of treatment systems, and social and immigration policies as the basis for racial/ethnic disparities in treatment. These issues are not in the TCU model and are not typically considered in research on treatment retention but are important in understanding inequities in treatment and will require larger social policy changes outside of the treatment system to address them.

Providers felt that recruiting bilingual staff and racially/ethnically diverse staff would help address disparities in retention. Providers’ Spanish proficiency is positively associated with treatment retention among adult Latinos in substance use treatment [36]. Providers in the present study said that for some of the youth they treat, family members were not fluent in English, impacting family engagement in treatment and youth retention. Therefore, providers should try to recruit treatment professionals that are fluent in languages spoken by families in their communities. In addition to language, recruiting and maintaining a culturally diverse workforce is one of the national CLAS standards and previous studies have found that clients of color who have psychotherapists who match their racial/ethnic backgrounds attend more psychotherapy sessions and are less likely to drop out of therapy [37, 38]. However, it is important to acknowledge that the behavioral health workforce in general is not racially/ethnically representative of the clients served and there is a call for initiatives to recruit underrepresented individuals of color into the behavioral health workforce to address this gap [39, 40]. Therefore, professional schools can play a role by recruiting a diverse student body that can adequately serve youth in treatment.

In addition to staffing and language, several of the other strategies that providers thought would help address disparities in retention match the CLAS national standards including training on culturally appropriate practices on an ongoing basis, and providing materials and signage in the languages used by the population in their service area. Providers acknowledged the need to examine racial/ethnic disparities in treatment retention further and find ways to address them. States and insurance companies could monitor disparities in retention in a similar way they monitor other performance measures and listen to providers and families on ways that any detected disparities can be eliminated. This might vary depending on the populations served and it is important that their voices be included. As the racial/ethnic diversity of youth is increasing, it is imperative that more attention be paid to understanding and addressing the needs of youth of color and immigrant families in research, practice, and policy.

This study adds to the limited literature on retention among adolescents in substance use treatment, and in the scant literature on racial/ethnic disparities in treatment retention among this age group. Few studies have included provider perspectives when examining retention barriers and facilitators. Providers are directly involved in the management and provision of services and can consider policy and management barriers and facilitators that may not be apparent through use of administrative data, or from the experiences of clients or their families. Providers’ perspectives are needed to ensure that any proposed interventions will have a better chance of successfully being implemented [41].

Some limitations should be mentioned. This study was conducted with providers in one state, and thus generalizability is limited. Massachusetts historically has had a strong focus on health care access and, therefore, it is likely that providers from other states might describe different barriers. As several providers mentioned, the state’s substance use agency and Medicaid program provided financial and non-financial supports that facilitated the provision of services that private health insurance plans did not. Although these characteristics may be specific to Massachusetts, they provide an example of changes at the systems levels that other states could explore as a mechanism to support treatment services for youth. Additionally, we interviewed providers from only 19 facilities. Although this number might seem small, there are few facilities that serve adolescents, particularly stabilization and residential facilities, in the state (or in most states). Finally, most of the providers included in this study were at the management level. Incorporating more clinicians and including other staff (e.g., intake coordinators) could offer additional insight into issues affecting treatment retention from a program’s perspective.

Future research should examine provider perspectives from providers in multiple states. Differences in the substance use workforce, adolescent demographics and types of substances used, and the organization, funding, and policies related to treatment in different states may shed light on additional factors that influence treatment retention and equity in retention among youth. Additionally, a larger number of providers could also allow for comparing perspectives in barriers, facilitators, and racial/ethnic disparities in retention by providers with different characteristics (e.g., management compared to clinicias, providers of color compared to White providers). Some of the feedback from providers also suggest new areas of inquiry. For example, new studies could examine the impact of health care polices and staffing issues on treatment retention among youth. Additional research is also needed to understand inequities in retention in general as there is a dearth of research in this area. Learning from providers and state substance use agencies who have been successful at addressing equity issues could inform the development of strategies that may be implemented more broadly to address this gap.

## Conclusions

Given the potential long-term negative consequences of substance use in adolescence and the benefits of longer treatment retention among youth who are in treatment, it is critical that adolescents who access substance use services remain in and complete treatment and that there is equity in treatment retention. Understanding barriers and facilitators to retention from multiple stakeholders' viewpoints is a necessary step in addressing this complex issue. This study offers the perspective of providers, a perspective that is seldomly incorporated in research in this area.

## Data Availability

The data generated during the current study are not publicly available to protection of confidentiality. The participant consent form stated that the data would not be shared with individuals outside the study team, except as required by law.

## Abbreviations

SUD: Substance Use Disorder
BSAS: Bureau of Substance Addiction Services
CLAS: Culturally and Linguistically Appropriate Services
NIATx: Network for the Improvement of Addiction Treatment
